# First person – Akash Gupta

**DOI:** 10.1242/bio.053066

**Published:** 2020-05-29

## Abstract

First Person is a series of interviews with the first authors of a selection of papers published in Biology Open, helping early-career researchers promote themselves alongside their papers. Akash Gupta is first author on ‘A novel and cost-effective *ex vivo* orthotopic model for the study of human breast cancer in mouse mammary gland organ culture’, published in BiO. Akash conducted the research described in this article while a PhD Scholar in Rajendra Mehta's lab at IIT Research Institute, Chicago, USA. He is now an assistant research scientist in the lab of Syreeta L. Tilghman at the University of Arizona, Department of Medicine, Tucson, USA, investigating drug efficacy modeling using human organoids culture for the treatment of cancers.


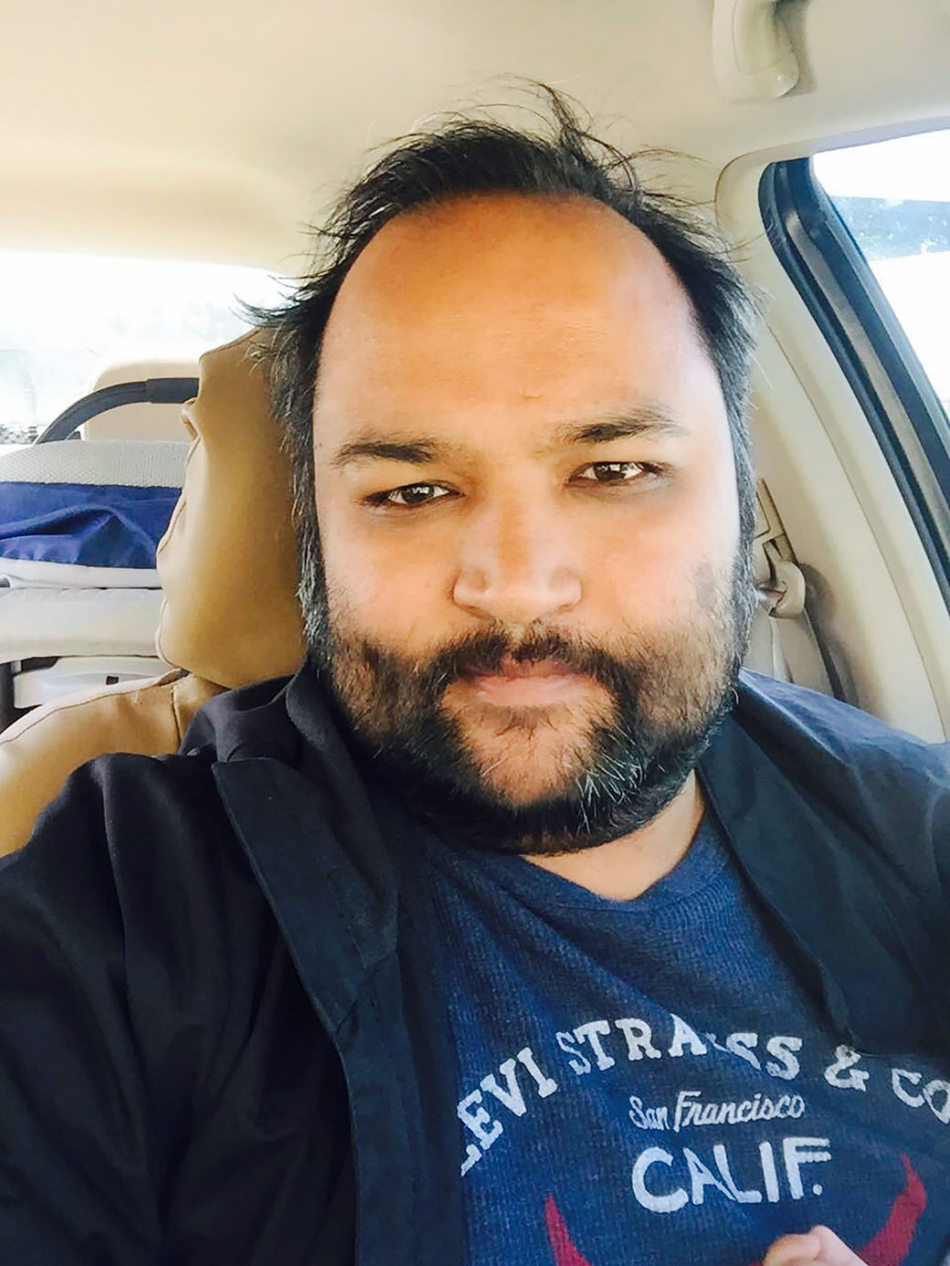


**Akash Gupta**

**What is your scientific background and the general focus of your lab?**

I finished my BSc in Microbiology and Master's in Biotechnology from the University of Madras, India. I earned a PhD in Biology from the Illinois Institute of Technology, Chicago. My mentor was Dr Rajendra R. Mehta and my PhD thesis title was ‘Development of Genetically Engineered Aromatase Overexpressing and Aromatase Inhibitor Resistant Breast Cancer Cell Lines to Study Possible Therapeutic Implications of a Novel Antiprogestin’. After successful completion of the PhD in Biology, I was offered a postdoctoral fellowship at the Robert Lurie Cancer Centre at Northwestern University in Chicago, under renowned surgical oncologist, Dr Seema Khan. While a postdoctoral fellow at Northwestern University, I learned various animal techniques like ovariectomy, kidney capsule implantation, and xenografts. Most importantly, I developed human primary breast organoids from reduction mammoplasty and mastectomy samples from human breast. I also measured the gene expressions in 3D culture models, which led to a publication in the Science Translational Medicine Journal. Currently, I am part of the lab as an assistant research scientist at the University of Arizona, which studies the microbiome and its association with the gut. I developed colon organoids culture from human colon and duodenum biopsies.

**How would you explain the main findings of your paper to non-scientific family and friends?**

Breast cancer is one of the major cancers diagnosed around the world. Breast cancer related deaths have been considerably reduced over the years, as there are many treatments available for advanced breast cancer. However, there is still a need for more studies addressing drug resistance. My work on the development of a novel model, breast cancer in mouse mammary organ culture (BCa-MMOC) can be utilized to test novel agents in a cost-effective lab setting; thus allowing researchers to study the efficacy of new drugs and also new targets for treatment.

**What are the potential implications of these results for your field of research?**

The results from my *ex vivo* mammary gland study will provide researchers with strategies and tools as an additional model and provide another option to study drug resistant forms of breast cancer. Breast cancer in BCa-MMOC provides a unique interface between *in vitro* and *in vivo* studies and allows researchers to systematically delineate the effect of the tumor microenvironment as well as delineating the molecular mechanism behind chemopreventive or chemotherapeutic compounds. This model will be a major asset in the decision-making process before designing expensive *in vivo* studies.

“This model will be a major asset in the decision-making process before designing expensive *in vivo* studies.”

**What has surprised you the most while conducting your research?**

It was interesting as well as surprising to mimic the pattern of breast cancer cell growth in the mouse mammary gland dissected from the thoracic abdominal region as it is observed in actual breast cancer in human. When exposed to estrogen and progesterone, the mouse mammary gland as an organ culture provides an equally nourishing and adequate growth environment as seen in *in vivo* animal xenograft studies. To me it was fascinating and comforting that I was able to establish this model and answer similar questions as those that utilize *in vivo* models.

**Figure:**
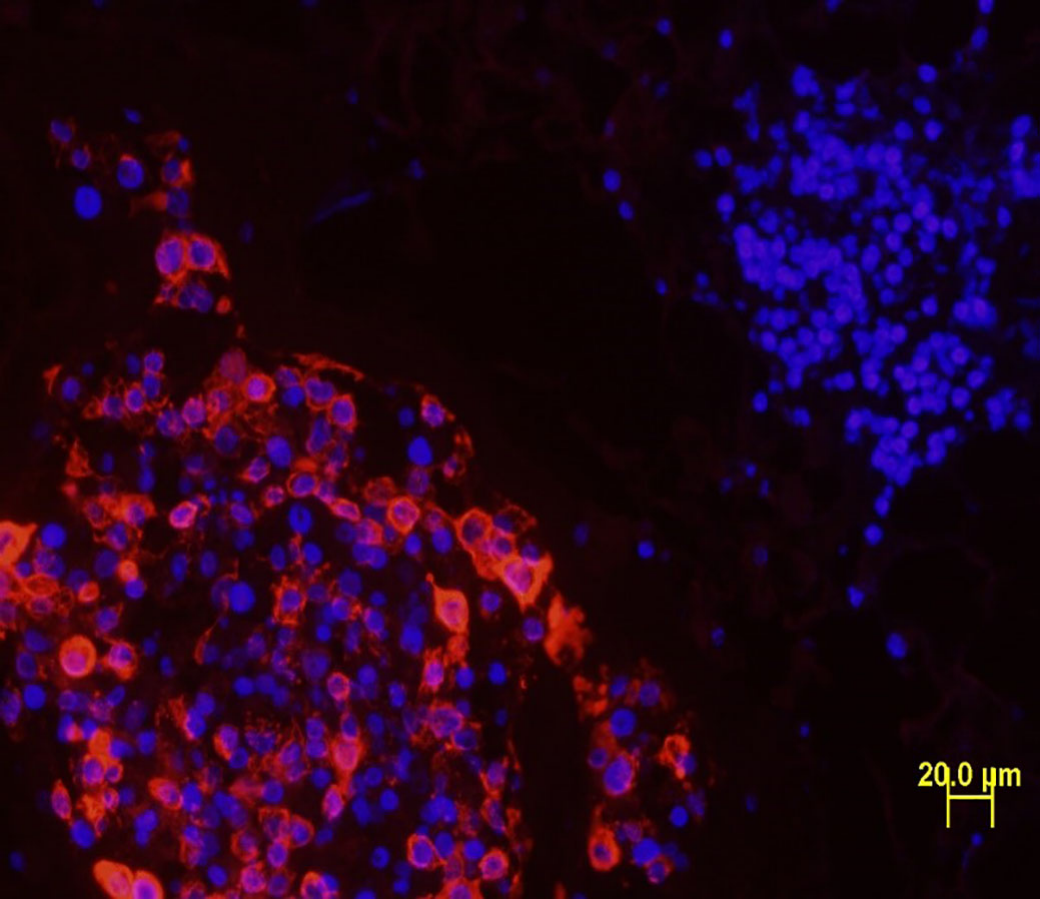
**Human breast cancer in BCa-MMOC.**

**What, in your opinion, are some of the greatest achievements in your field and how has this influenced your research?**

Discovery of the role of the aromatase enzyme and its inhibitors and several other therapeutic compounds that target estrogen receptors in breast cancer are some of the greatest achievement for hormone dependent breast cancers. I admire the late Dr Angela Brodie for her work on aromatase and aromatase inhibitors, which helped guide me through my PhD thesis. I was in awe when I met her briefly during a San Antonio Breast Cancer Symposium meeting in 2012. I am incredibly thankful to Dr Syreeta L. Tilghman, who was mentored by Dr Brodie in her early career, for giving me the opportunity to collaborate and present my research.

**What changes do you think could improve the professional lives of early-career scientists?**

As acquisition of extramural funding is becoming more challenging, I am worried that talented young scientists may be unable to conduct competitive research. I would like to remind all early career scientists to focus on what they want to achieve to pursue their goals. It is critical to choose mentors who can enhance your careers. It is critical to publish original research in a timely fashion and consider lower impact journals instead of only considering journals with high impact factors. This will aid you in being more visible to the research community while building a prolific research career.

“As acquisition of extramural funding is becoming more challenging, I am worried that talented young scientists may be unable to conduct competitive research.”

**What's next for you?**

I am looking for a junior faculty position at an academic institution.
